# Progress and Challenges of InGaN/GaN-Based Core–Shell Microrod LEDs

**DOI:** 10.3390/ma15051626

**Published:** 2022-02-22

**Authors:** Johanna Meier, Gerd Bacher

**Affiliations:** Werkstoffe der Elektrotechnik and CENIDE, Universität Duisburg-Essen, Bismarckstraße 81, 47057 Duisburg, Germany; johanna.meier@uni-due.de

**Keywords:** GaN, core–shell, microrod, nanowire, LED

## Abstract

LEDs based on planar InGaN/GaN heterostructures define an important standard for solid-state lighting. However, one drawback is the polarization field of the wurtzite heterostructure impacting both electron–hole overlap and emission energy. Three-dimensional core–shell microrods offer field-free sidewalls, thus improving radiative recombination rates while simultaneously increasing the light-emitting area per substrate size. Despite those promises, microrods have still not replaced planar devices. In this review, we discuss the progress in device processing and analysis of microrod LEDs and emphasize the perspectives related to the 3D device architecture from an applications point of view.

## 1. Introduction

InGaN/GaN-based core–shell nano- and microrods for light-emitting diode (LED) applications have been a vivid research topic in recent years [1,2,3]. The unique three-dimensional (3D) architecture promises novel properties that may be attractive for solid-state lighting or even open further application fields of GaN-based LEDs. These include (flexible) white LEDs [3,4], single-photon sources [5], and high-frequency LEDs [1,6]. Microrod LEDs have a high surface-to-volume ratio, creating an enlarged light output area for the same chip size [7]. Additionally, the small footprint reduces the defect density, and the 3D structure gives access to different crystal facets [8].

Due to the wurtzite lattice, spontaneous and piezoelectric polarization that depends on the crystal facet occurs in InGaN/GaN heterostructures due to crystal anisotropy. The polarization-induced electric field leads to the quantum confined Stark effect (QCSE) [9,10]. This causes a bending of the band structure in the active region, resulting in a reduced overlap of the electron and hole wave function [11] and, hence, in a reduced radiative recombination rate and enhanced charge carrier lifetimes [12]. The electric field is particularly strong in the c-direction, in which planar InGaN/GaN LEDs are usually grown [13]. In contrast, the dominating facet in microrod LEDs is the m-plane, also called the nonpolar plane, where the QCSE is absent. Utilizing the nonpolar plane of microrods offers great advantages, e.g., higher modulation speed or reduced operating currents [14], of microrod LEDs.

The challenge to grow vertical 3D structures rather than planar devices was mastered using different strategies, including catalyst-assisted [15,16] and catalyst-free [17,18] approaches. Novel contacting schemes as well as advanced characterization tools [2] had to be developed due to the complex 3D architecture of microrod LEDs. Meanwhile, the first blue, green, red, and white microrod LEDs have been reported [3,19,20,21]. Furthermore, high-frequency LEDs based on microrod structures have been demonstrated [1,6].

This review focuses on bottom-up, core–shell InGaN/GaN LEDs. First, the properties of the microrods and the associated crystal facets are discussed. Subsequently, core–shell InGaN/GaN LED devices are introduced, and characteristic device properties are emphasized. Lastly, selected application fields of microrod LEDs are outlined.

## 2. Crystal Facets of Me-Polar and N-Polar InGaN/GaN Core–Shell Microrods

**Figure 1:**
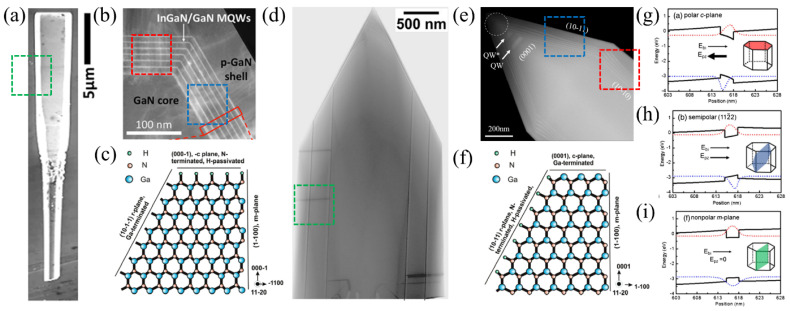
(**a**) SEM image of N-polar microrod. Adapted with permission from Ref. [20]. Copyright 2018 American Chemical Society. (**b**) Cross-sectional STEM-HAADF image of the tip of N-polar microrod. Adapted with permission from Ref. [20]. Copyright 2018 American Chemical Society. (**c**) Schematic GaN atomic structure in [0001] direction. Reprinted with permission from Ref. [22]. Copyright 2011 American Chemical Society. (**d**) Bright-field STEM image of Me-polar microrod. Adapted with permission from Ref. [3]. Copyright 2016 WILEY-VCH Verlag GmbH & Co. KGaA, Weinheim. (**e**) STEM image of Me-polar microrod. Adapted with permission from Ref. [24]. Copyright 2018, Robin et al. (**f**) Schematic GaN atomic structure in [000$\bar{1}$] direction. Reprinted with permission from Ref. [22]. Copyright 2011 American Chemical Society. Simulated band diagram for a single quantum well (In_0.2_Ga_0.8_N (3 nm)/GaN (15 nm)) grown on the (**g**) c-plane, (**h**) semipolar (11$\bar{2}$2) plane, and (**i**) nonpolar m-plane. Reprinted with permission from Ref. [11]. Copyright 2018 The Optical Society. The red and blue dotted lines illustrate the electron and hole wave functions, respectively.

InGaN/GaN core–shell microrods can be categorized into two approaches, depending on the growth direction: metal-polar (Me-polar) and nitrogen-polar (N-polar) [22,23] structures. Me-polar microrods are grown in the [0001] direction, while N-polar microrods are obtained by growing in the [000$\bar{1}$] direction. The polarity changes the morphology of the microrods significantly. In Figure 1, scanning electron microscope (SEM) images and scanning transmission electron microscope (STEM) images are shown of N-polar and Me-polar microrods.

The polarity can be controlled by the growth substrate and/or different buffer layers. Common growth substrates for core–shell InGaN/GaN microrods are sapphire or Si(111). Sapphire as commonly used is a polar substrate, and, combined with an n-GaN buffer layer [21], it allows successful growth of microrods with homogeneous polarity [25]. Si(111) as a growth substrate has a high thermal and electrical conductivity, but it is a nonpolar material. Hence, GaN-based microrods will grow in mixed polarities (Me-polar and N-polar) on Si(111) [26]. Additionally, the direct growth of GaN on Si(111) is not possible due to the Ga melt-back etching effect. An AlN buffer layer on Si-pillars can help to control the surface polarity of the microrods and reduce the Ga melt-back etching effect [27,28].

N-polar microrods (Figure 1a) have a flat c-facet tip with large m-facets on the side. The semipolar facet, as illustrated in Figure 1b, is comparatively small. The atomic crystal structure (see Figure 1c) shows that the nitrogen atoms on the tip surface are passivated by hydrogen. Hence, the c-plane of N-polar microrods is stable and has a low growth rate [22]. In contrast, the r-facets are terminated by Ga atoms, which can be altered by hydrogen atoms. This leads to small r-facets and stable large m-facets [29]. A thickness gradient is observed along the m-plane, which is usually assigned to a diffusion gradient of the In atoms [29]. A STEM image of Me-polar microrods is shown in Figure 1d. Quantum wells (QWs) on the semipolar facet (Figure 1e) are usually thinner than on the nonpolar facet [2]. A thickness gradient of the nonpolar QW is also measured for Me-polar microrods [2,24]. Me-polar microrods have N-terminated and H-passivated r-facets (Figure 1f), causing stable r-planes. As a result, Me-polar microrods have a pyramidic tip [22].

The different facets lead to a different band structure that influences the electro-optical behavior of InGaN/GaN core–shell microrod LED. Due to the lack of crystal symmetry, the total polarization differs on the various facets. Hence, the band structure of the active QW region also changes.

The band structure depicted in Figure 1g–i and the respective electron- and hole-wave functions were calculated for different crystal planes solving the one-dimensional Schrödinger–Poisson equation including the drift-diffusion model [11]. The calculations show a p-GaN (15 nm), In_0.2_Ga_0.8_N (3 nm), n-GaN (15 nm) single quantum well (SQW) of an LED at 100 A/cm^2^. The electric field from the spontaneous and piezoelectric polarization is summarized as E_pz_ and the built-in field from the p–n-junction is denoted as E_bi_. The band structure of the c-plane, also called polar plane, is influenced by a strong polarization field E_pz_ and a weaker built-in field E_bi_ of opposite direction. The electron (hole) wave function shifts toward the p (n) region, which decreases the electron–hole overlap and, thus, results in a reduced radiative recombination rate. The semipolar planes (10$\bar{1}\bar{1}$) and (20$\bar{2}\bar{1}$)) behave qualitatively similar as the polar c-plane. E_bi_ and E_pz_ are in opposite directions, but E_pz_ is much weaker. In contrast, E_pz_ of the semipolar planes (20$\bar{2}$1) and (11$\bar{2}$2) points in the same direction as E_bi_. This also leads to a separation of electrons and holes, but in the opposite direction to that described above. In case of the m-plane, however, the polarization field E_pz_ is absent, and only the built-in field E_bi_ influences the band structure. Hence, the overlap of the electron and hole wavefunction is largest, leading to enhanced recombination rates (see Figure 1f).

Thus, Me-polar and N-polar microrods offer the accessibility of different crystal facets, which tremendously influence the band structure of the active region. Hence, the different crystal facets are expected to have a huge impact on the electro-optical behavior of InGaN/GaN microrod LEDs.

## 3. InGaN/GaN Core–Shell Microrod LEDs

As outlined above, the N- or Me-polar growth of microrods on various substrates results in a slightly different morphology that influences the LED device concept. In addition, InGaN/GaN microrods have been grown using catalyst-assisted and catalyst-free approaches. The vapor liquid solid (VLS) method uses metal droplets (Fe, Ni, Au) as a catalyst, usually in a metalorganic chemical vapor deposition (MOCVD) process. Blue-emitting core–shell InGaN/GaN microrod LEDs have been successfully realized using this technique [15,16,30,31,32,33]. The main drawback is the contamination of the crystal by the metallic catalyst. Furthermore, this approach leads to microrods with various sizes and crystallographic orientations, which hampers processing of efficient LEDs.

The catalyst-free approach can use molecular beam epitaxy (MBE) or MOCVD. Since core–shell structures are more challenging to be realized by MBE due to the shadow effect resulting from the width of the microrods [34], we focus on MOCVD-grown microrod LEDs in this review. The catalyst-free approach can be categorized into self-assembled growth and selective area growth (SAG). Self-assembled growth leads to statistical formed nucleation centers on the substrate [18]. Hence, the microrods have a broad distribution in size, height, and position. In the SAG process, microrods are grown in openings of a dielectric mask (e.g., SiO_x_ or SiN_x_) on the substrate [25,35]. The growth can be precisely controlled by the spacing and the size of the openings. Even mask-less, site-controlled growth is possible using nanoimprint lithography [17].

**Figure 2:**
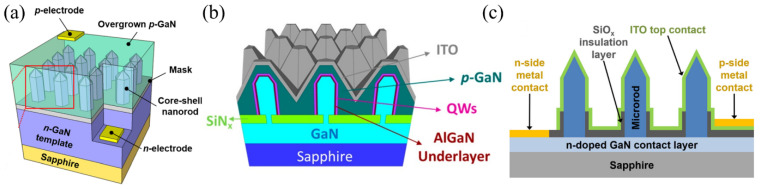
Schematic microrod chip structures with different top contact solutions. (**a**) Coalescent p-GaN. Reprinted with permission from Ref. [37]. Copyright 2016, Jung et al. (**b**) Coalescent p-GaN and ITO. Reprinted with permission from Ref. [1]. Copyright 2019 American Chemical Society. (**c**) ITO. Reprinted with permission from Ref. [3]. Copyright 2016 WILEY-VCH Verlag GmbH & Co. KGaA, Weinheim.

Depending on the substrate and on the microrod morphology, different device concepts are reported in the literature. Typically, the n-GaN core needs to be contacted via the substrate. Microrods grown on conductive Si(111) can easily be contacted by a back contact (e.g., Ni/Au) [6,32]. For microrods grown on sapphire, the n-contacts are deposited on the sapphire/ n-GaN buffer layer as seen in Figure 2a. Beforehand, the microrods and part of the buffer layer are etched with the help of a photolithography process to access the n-GaN template [3].

The main challenge is to contact the p-GaN, which suffers from low conductivity and has a 3D structure. A transparent conductive oxide (TCO), such as indium tin oxide (ITO), is a standard transparent p-contact layer to achieve lateral current spreading in planar InGaN/GaN-based LEDs. A transparent current spreading layer is also required for a uniform current injection into the microrod LEDs. In case of ITO deposition, the pillar shape of the microrods can lead to a thin ITO layer with a high resistance at the microrod bottom [36]. Figure 2 illustrates different approaches for the device architecture and the p-contact definition.

One approach is the planarization of the device by coalescing the microrods [19,37] as seen in Figure 2a. For a reduced contact resistance, ohmic Ni/Au contacts are directly placed on the p-GaN with no additional current spreading layer. The drawback of the coalescent p-GaN is an enhanced surface roughness and a high resistivity that leads to inhomogeneous emission characteristics due to current leakage paths from the p-electrode through the p-GaN to the tip of the microrods [19]. Another possibility to planarize the microrods is to fill the space with an insulating spin-on-glass (SOG) by spin-coating and subsequently etching the SOG at the microrod tips, where the ITO is deposited [24]. Since the SOG insulates the microrod sidewalls, the current spreading layer only performs at the microrod tips. Hence, a homogeneous contacting of the microrods is not guaranteed.

An alternative strategy (Figure 2b) involves semi-coalescent microrods, creating a pyramid-shaped p-GaN around the core and the active region [1,14]. This offers the advantage of a more homogeneous ITO layer that can be deposited on top for current spreading. The gold contacts on the ITO layer are usually designed as a finger structure, since the conductivity of the ITO is limited [3]. Most device concepts are based on ITO deposition across the whole microrod structure (as seen in Figure 2c). The shadowing effect of the microrods can be reduced by enhancing the working distance in the sputtering process. A uniform thick ITO layer leads to a homogeneous emission across the whole LED chip [3,36].

A different device concept on the lab scale is a single microrod device, where a single microrod is individually contacted. In most cases, the single microrod is placed on an insulating substrate, and then the p-shell and n-core are contacted separately with e-beam lithography [20,29,30,38,39].

**Figure 3:**
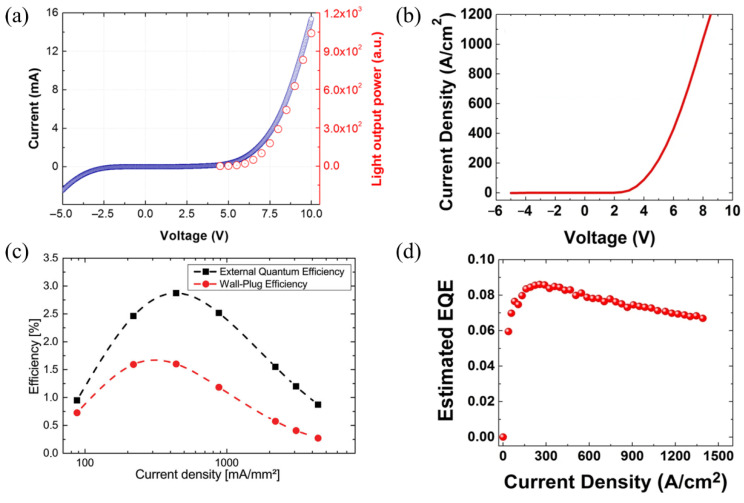
(**a**) I–V characteristic (blue) and light output power (red) of a microrod LED with coalescent p-GaN. Adapted with permission from Ref. [37]. Copyright 2016, Jung et al. (**b**) I–V characteristic of a blue-emitting microrod LED. Adapted with permission from Ref. [14]. Copyright 2018, Nami et al. (**c**) Wall-plug efficiency (red) and EQE (black) of a microrod LED versus current density. Adapted with permission from Ref. [3]. Copyright 2016 WILEY-VCH Verlag GmbH & Co. KGaA, Weinheim. (**d**) Estimated EQE versus current density of a microrod LED. Reprinted with permission from Ref. [14]. Copyright 2018, Nami et al.

Figure 3a depicts the current–voltage (I–V) characteristic and the light output power of a microrod LED with coalescent p-GaN (see Figure 2a) [37]. A correlated increase in light output power and current is observed with increasing forward voltage. The device exhibits a strong leakage current at reverse bias with a rectifying behavior < 1 at ±5 V. Distinct leakage currents and insufficient rectifying behaviors have been observed in several microrod LEDs. Table 1 lists some rectifying values of InGaN/GaN microrod LEDs. Rectifying values at ±5 V of <10 are published [3,19,40], as well as values of 10 [21] and 17 [41]. Figure 3b shows the I–V characteristic of a microrod LED (device architecture similar to Figure 2c with a nice rectifying behavior of I^+^_(5 V)_/I^−^_(5 V)_ = 85 and low leakage current. Such a pronounced diode-like I–V characteristic has been achieved in a few microrod LEDs with device architectures according to Figure 2b,c [14,21], reaching rectifying values of I^+^_(5 V)_/I^−^_(5 V)_ = 153 [21].

The internal quantum efficiencies (IQEs) for microrods improved in the recent years from ~8% [43] to a record value of 62% [14] at room temperature [19,21,44]. The IQE at low excitation is influenced by nonradiative losses, while, at high excitation conditions, the droop phenomenon occurs [14]. Spatially resolved IQE measurements show a higher IQE at the bottom than at the tip, attributed to a higher In-content and, hence, a higher defect density close to the tip [44,45].

Despite the high IQE, microrod LEDs still suffer from relatively low external quantum efficiencies (EQEs). Figure 3c shows the wall-plug efficiency and the EQE of the device seen in Figure 2c [3]. The device has a maximum wall-plug efficiency of 1.5% at 30 A/cm^2^, while the maximum EQE is equal to 3% at 40 A/cm^2^. As shown in Figure 3d, the EQE varies with current density, with a maximum value of 8.3% at 260 A/cm^2^ [14].

Table 1 lists the efficiencies of published absolute EQE values that range from 0.63% up to 12.6%. Values of EQE = 8.3% [14] (see Figure 3d) and EQE = 10% [21] have been achieved at relatively high current densities. An EQE value of 12.6% has been achieved at a current density of 14.4 A/cm^2^. Here, the current density was calculated by taking into account the radial geometry of the quantum wells [41]. White flexible phosphor-converted microrod LEDs reach an EQE of almost 10% [41].

## 4. Electrooptical Analysis of Microrod LEDs

**Figure 4:**
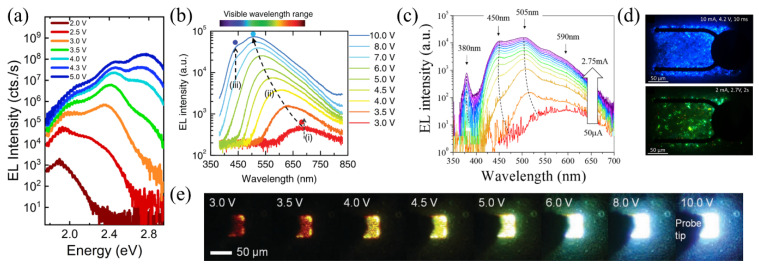
(**a**) EL spectra of a microrod LED with ITO as current spreading layer at different voltages on a logarithmic scale. Reprinted with permission from Ref. [46]. Copyright 2021 AIP Publishing. (**b**) EL intensity over wavelength of a microrod-embedded LED at different voltages. Reprinted with permission from Ref. [19]. Copyright 2011 WILEY-VCH Verlag GmbH & Co. KGaA, Weinheim. (**c**) Logarithmic EL spectra of a microrod LED on Si(111) planarized with spin-on-glass at various injection currents. Reprinted with permission from Ref. [24]. Copyright 2018, Robin et al. (**d**) EL images of a microrod LED chip at 2 mA (top) and 10 mA (bottom). Reprinted with permission from Ref. [3]. Copyright 2016 WILEY-VCH Verlag GmbH & Co. KGaA, Weinheim. (**e**) EL images of nanorod-embedded LED at different voltages. Reprinted with permission from Ref. [19]. Copyright 2011 WILEY-VCH Verlag GmbH & Co. KGaA, Weinheim.

Characteristic electroluminescence (EL) spectra have been reported for different microrod LEDs. A blue shift of the dominant EL emission with rising voltage or injection current was detected in microrod LEDs on sapphire [42] and on Si(111) [24] substrates, for both self-assembled [38] and SAG [21] microrod LEDs. Figure 4 shows EL spectra from different microrod LEDs, as well as optical images of operating microrod LEDs.

The EL spectra visualized in Figure 4a–c stem from devices with different p-GaN contact layout. The microrod LED, with the EL spectrum shown in Figure 4a, is grown on sapphire and has ITO as a current spreading layer. Figure 4b shows the EL spectra from a microrod-embedded LED on sapphire, while the EL spectra depicted in Figure 4c are from a microrod LED on Si(111) planarized with SOG combined with ITO. All EL spectra change strongly with rising voltage or current; the EL spectra broaden and shift to higher energies. In addition, operating the LEDs at high injection conditions gives rise to distinct EL peaks. Depending on the device architecture and the operation conditions, the EL spectra can cover the whole visible range. While some groups detect red luminescence (690 nm) at low operating conditions [19,46], others show EL spectra starting at a peak wavelength of 500 nm [1,3,37] or 590 nm [24] at low excitation. Additionally, the literature reports a slight shift of the individual EL peaks with driving voltage/current.

Since the EL spectra cover a wide spectral range, it is assumed that the various EL peaks stem from different facets of the microrods. Regions with higher In content and/or increased quantum well thickness result in low energy emission and low turn-on voltages due to their smaller band gap. The red and green emissions are usually attributed to the polar and semipolar planes, respectively, while the blue emission mainly stems from the nonpolar plane. The slight energy shift of the individual EL peaks is presumably due to the screening of the QCSE and/or the band filling effect. The strongest shift is observed for the c-plane quantum well, while m-plane quantum wells exhibit a much smaller energy shift with operation voltage or driving current due to the absence of the polarization fields [24]. Figure 4d,e show images of an operating microrod LED at different voltages. Figure 4d visualizes a microrod LED on a sapphire substrate with an ITO based p-contact (see Figure 2c). The microrods of Figure 4e are embedded into a p-GaN matrix (see Figure 2a). A homogeneous emission is visible across the LED chip, and the overall color changes with operation voltage.

**Figure 5:**
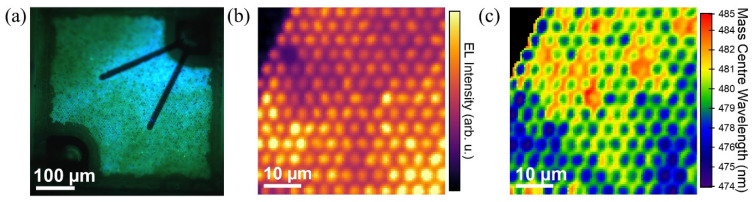
(**a**) EL image of a microrod LED at 4 V. Reprinted with permission from Ref. [46]. Copyright 2021 AIP Publishing. (**b**) Maximum EL intensity map of microrod LED at 3.6 V [47]. (**c**) Corresponding mass center wavelength map at 3.6 V [47].

Micro-EL (µEL) spectroscopy can visualize tiny changes in the electroluminescence across a chip. Figure 5 shows an optical image of a microrod LED at 4 V and corresponding µEL maps at 3.6 V. The device uses ITO as a current spreading layer and is similar to that shown in Figure 2c. Microrods that are close to the contact fingers emit more bluish, and microrods with some distance to the contacts appear greenish. This is attributed to the finite conductivity of the ITO and the corresponding lateral voltage drop; microrods closer to the contacts experience a higher bias, while, at some distance to the contacts, only microrod regions with a higher In content and, thus, a lower bandgap and a smaller turn-on voltage are able to emit EL. The µEL map of an area of 50 × 50 µm^2^ at a voltage of 3.6 V shows additional inhomogeneities in the EL intensity. Figure 5b,c show the corresponding maximum EL intensity and the mass center wavelength, respectively, of the device. The p-contact finger is at the lower border of the figure. Microrods closer to the contact exhibit a higher EL intensity and a lower emission wavelength due to higher voltages close to the contacts. Thus, the variations in the bandgap of the different active areas of the microrods that lead to multiple EL peak also cause inhomogeneities in both color and EL intensity across the microrod LED chip.

**Figure 6:**
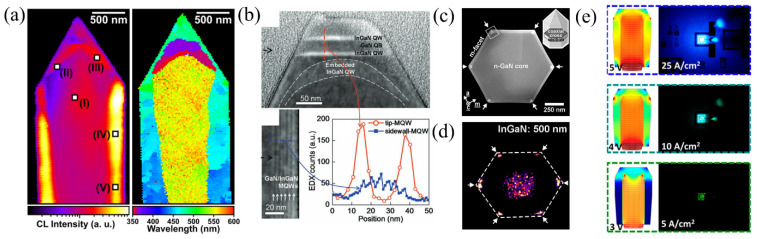
(**a**) CL intensity image (left) and correlated CL peak wavelength image (right) of a microrod. Reprinted with permission from Ref. [2]. Copyright 2016 American Chemical Society. (**b**) TEM images of the InGaN/GaN MQWs on top (top) and on the side (bottom, left) of a microrod. The inset shows an EDX profile of the In content. Reprinted with permission from Ref. [19]. Copyright 2011 WILEY-VCH Verlag GmbH & Co. KGaA, Weinheim. (**c**) HAADF image of a coaxial cross-section of a single microrod. The inset shows a schematic microrod including the cross-section. Reprinted with permission from Ref. [54]. Copyright 2018, Schmidt et al. (**d**) Monochromatic CL intensity image at 18 K and 500 nm of the cross-section. Reprinted with permission from Ref. [54]. Copyright 2018, Schmidt et al. (**e**) Simulation of the current injection from 3–5 V showing the regions of the microrod contributing to the EL spectrum (left) and corresponding EL images (right) of the microrod LED. Reprinted with permission from Ref. [1]. Copyright 2019 American Chemical Society.

For a microscopic analysis of the microrod active region, photoluminescence (PL) mapping [44,46,48] and cathodoluminescence (CL) measurements [16,21,40,45,49,50,51,52,53] were performed by different groups to gain insight into the various spectral contributions of the EL. Cathodoluminescence (CL) uses an electron beam scanning the sample and generating electron–hole pairs which recombine. Due to the focused electron beam, CL spectroscopy has a very high spatial resolution. In the CL map shown in Figure 6a, the highest emission intensity stems from the m-planes. Additionally, the CL map shows a blue shift from tip to the bottom on the nonpolar facet [2]. The semipolar facets emit with a higher wavelength and show a smaller QW thickness compared to the nonpolar facets. Thus, the wavelength shift was mainly attributed to a nonuniform In incorporation into the QW due to reduced gas diffusion between the microrods. Additionally, it has been shown that In incorporation varies for different facets due to anisotropic surface formation energies. This influences the diffusion of the adatoms [19], leading to varying growth rates on the facets. Hence, the semipolar facet reveals a thinner QW than the nonpolar one, and the QW at the polar facet is the thickest. Additionally, Hong et al. demonstrated that the In content was four times higher at the tip MQWs than at the sidewalls (see Figure 6b). MQWs with such a high In content are the origin for the red EL.

An even more detailed characterization was reported by Müller et al. performing CL in a scanning transmission electron microscope (STEM) [2]. Additionally, the microrods were analyzed with high-angle annular dark field (HAADF) contrast in the STEM to correlate the optical characteristics with the real crystal structure. The HAADF STEM data show an increase in the In content from 11% at the bottom to 13% at the tip of the microrod, while the QW thickness changes from 6 nm to 13 nm [2]. Furthermore, self-organized triangular nanoprisms with high In content were found at the edges (see Figure 6c) [54,55], creating emission centers for green luminescence (see Figure 6d).

Nami et al. simulated the current distribution in microrod LEDs to complete the picture of the color-shifting EL and the In accumulation and distribution in the microrods [1]. The simulation showed that the current is distributed from the top to the sidewalls. As mentioned above, the c-plane at the top exhibits QWs with higher In content. Hence, the microrod emits at higher wavelengths at low operation voltages. At higher voltages, the nonpolar sidewalls are excited. The thinner QWs with less In content lead to a blue-shift of the LED.

There are a few approaches demonstrated in the literature to control the emission color of microrod LEDs. For example, it was demonstrated that an AlGaN spacer between each QW can suppress In decomposition of the InGaN QW and, hence, limit the shift of the EL spectrum with increasing current [56]. It has also been shown that the EL can be influenced by contacting only particular parts of the microrod [51] or by a dielectric passivation [42]. With this approach, only emission originating from the m-plane can be achieved. Zhang et al. introduced a post-growth treatment [40]; after processing a full microrod LED, the ITO and Mg-doped GaN (p-GaN) were etched at the tip via RIE (called “passivation”). Therefore, only the sidewalls were contacted, and only the m-plane QWs contributed to the EL.

## 5. Application of InGaN/GaN Core–Shell Microrod LEDs

The high aspect ratio of the microrods offers great possibilities for future core–shell InGaN/GaN microrod LED devices. The increased active area compared to planar devices with the same substrate sizes is highly attractive for the application of microrod LEDs in solid-state lighting [3,21,41], as it gives the possibility of operating the microrod LEDs at a reduced current density, thereby avoiding the droop phenomenon. Additionally, the nonpolar sidewalls enable prospects beyond solid-state lighting. The nonpolar sidewalls reduce the radiative recombination lifetime and, thus, enhance the modulation frequency of microrod LEDs [1,6]. This extends the application field to display technologies and visible-light communication (VLC). Microrods also enable the development of flexible LEDs for use in bendable displays or as curved light sources [41].

**Figure 7:**
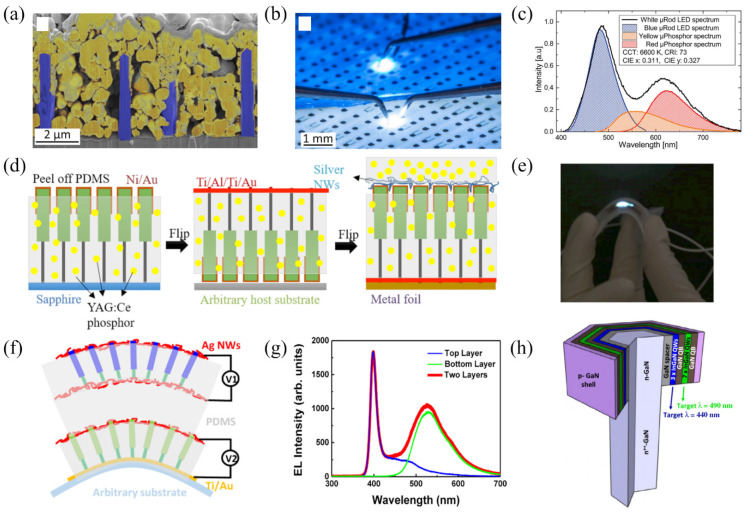
(**a**) Colored SEM image of a microrod LED (blue) where a microcrystalline phosphor (yellow) was deposited between the microrods. Reprinted with permission from Ref. [3]. Copyright 2016 WILEY-VCH Verlag GmbH & Co. KGaA, Weinheim. (**b**) Image of a microrod device with blue (top) and phosphor-converted white (bottom) emission. Reprinted with permission from Ref. [3]. Copyright 2016 WILEY-VCH Verlag GmbH & Co. KGaA, Weinheim. (**c**) EL spectra of the white microrod LED and fits of the individual components from the microrod LED and the phosphor. Adapted with permission from Ref. [3]. Copyright 2016 WILEY-VCH Verlag GmbH & Co. KGaA, Weinheim. (**d**) Fabrication process of a flexible white LED. Reprinted with permission from Ref. [41]. Copyright 2016 American Chemical Society. (**e**) Picture of a bended white LED at an injection current density of 3.9 A/cm^2^. Reprinted with permission from Ref. [41]. Copyright 2016 American Chemical Society. (**f**) Schematic of a dual color flexible LED with two sets of stacked microrods. Reprinted with permission from Ref. [57]. Copyright 2015 American Chemical Society. (**g**) EL spectra of the top and bottom LED and the combination of both emissions. Reprinted with permission from Ref. [57]. Copyright 2015 American Chemical Society. (**h**) Schematic of a heterostructure containing MQWs with different In content for blue and green emission. Reprinted with permission from Ref. [58]. Copyright 2021 Kappor et al. Advanced Photonics Research published by Wiley-VCH GmbH.

In this section, we discuss the successful fabrication of white and flexible microrod LEDs or high-frequency LEDs using InGaN/GaN core–shell microrod LEDs. Selected examples of the progress in white and/or flexible microrod LEDs are summarized in Figure 7.

State-of-the-art white LEDs consist of a blue-emitting InGaN/GaN LED and a phosphor such as cerium-doped yttrium aluminum garnet (YAG:Ce) for color conversion [59]. The micrometer-sized phosphor grains are usually embedded into silicone. Schimpke et al. embedded such a phosphor in a microrod array (see Figure 7a). Yellow- and red-emitting micrograin phosphors were used to convert the blue emission of the microrod LED and, thus, fabricate a white-emitting LED chip (see Figure 7b). The corresponding EL spectrum is shown in Figure 7c with the blue contribution of the microrod LED and the broad yellow–red spectrum of the micro-phosphor. In this way, the first microrod phosphor converted LED was realized with a coordinated color temperature (CCT) of 6600 K and a color rendering index (CRI) of 73.

Flexible white LEDs are currently dominated by organic LEDs (OLEDs) [60]. They can already be manufactured but have long-term stability issues since the emitting organic molecules are sensitive to the atmosphere. State-of-the-art InGaN/GaN LEDs have high efficiencies, as well as high lifetimes [61,62]. Following flexible blue and green microrod LEDs based on nitrides [63,64,65,66], in 2016, the first flexible white phosphor-converted LED based on microrods was demonstrated [41]. Figure 7d shows microrods embedded into a polydimethylsiloxane (PDMS) matrix mixed with a YAG:Ce phosphor. As a contact layer, silver nanowires were chosen, which are transparent, as well as flexible. The devices exhibited no degradation after multiple bending cycles. The first flexible phosphor-converted white microrod LED is depicted in Figure 7e; it had a CCT of 6306 K, a CRI of 54, and an EQE of 9.3%. The CRI was later improved to a value of 65 by using two layers of yellow and orange phosphors [4].

One approach to realize phosphor-free white LEDs is generating white light by combining red, green, and blue (RGB) luminescence. Microrods offer new possibilities to achieve multiple colors in one device. Combining the active regions of structures with different In content can generate a broad EL which is one possible approach toward white luminescence. Figure 7f shows the concept of stacking two microrod arrays emitting at different colors [57]. A semi-transparent green LED at the bottom is integrated with a transparent blue LED on top. Similar to the approach shown in Figure 7d the microrods were embedded into a polymer matrix for flexibility. The corresponding EL spectra are shown in Figure 7g. Operating either one or the other microrod array results in green or blue emission, respectively. Dual emission from both layers is achieved when operating both layers at the same time. Alternatively, Kapoor et al. generated dual-color emission from one microrod by combining QWs with different In content into one microrod [58] (see Figure 7h).

Another attractive application field for microrod LEDs is optical communication. Visible-light communication (VLC) uses light with wavelengths between 380 nm to 780 nm for wireless data communication. Information is transmitted by modulating the light at high frequencies. Using GaN-based LEDs, VLC can, in principle, be combined with solid-state lighting in our everyday life. A drawback of standard planar GaN-based LEDs is represented by the long electron–hole lifetimes due to the QCSE, which limit the modulation speed. Therefore, microrod LEDs with their nonpolar sidewalls are a great alternative.

**Figure 8:**
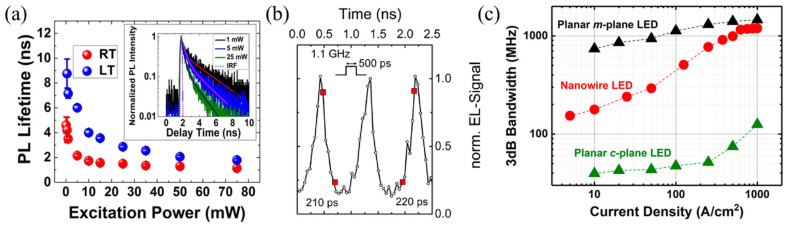
(**a**) PL lifetimes versus excitation power at room temperature (red, RT) and low temperature (blue, LT) from InGaN/GaN core–shell microrods. The inset shows the time-resolved PL decay curves for different excitation powers. Reprinted with permission from Ref. [14]. Copyright 2018, Nami et al. (**b**) Time-resolved EL signal of a microrod LED on Si(111). The devices were excited with a voltage pulse of 500 ps in width and a frequency of 1.1 GHz. Reprinted with permission from Ref. [6]. Copyright 2019 American Chemical Society. (**c**) The −3 dB modulation bandwidth versus current density for a microrod LED (red), a planar polar LED (green), and a planar nonpolar LED (black). Reprinted with permission from Ref. [1]. Copyright 2019 American Chemical Society.

PL lifetimes of InGaN/GaN core–shell microrods reported in the literature range from 20 ps to a few ns at room temperature. Figure 8a shows the charge carrier PL lifetime from an InGaN/GaN core–shell microrod LED as extracted from time-resolved photoluminescence (TRPL) measurements (shown in the inset). This measurement shows measurements on multiple microrods; hence, a PL signal stemming from different microrods and their various facets is recorded. The PL lifetime at low temperature is higher than at room temperature due to reduced nonradiative losses in the low-temperature regime [14]. Importantly, a decrease in PL lifetime with increasing excitation power is seen. This can be attributed to a screening of the QCSE and, thus, an increasing overlap of the electron and the hole wavefunction in the polar and semipolar QWs at higher excitation densities. TRPL measurements on the m-plane QW of a microrod show short lifetimes that are independent of the excitation density due to the absence of the QCSE [6].

In 2015, the first high-speed microrod LED was realized on a Si(111) substrate grown via a self-assembled and catalyst-free MOCVD process [6]. The devices showed EL signals under GHz excitation with 90–10% rise and fall times of about 220 ps (see Figure 8b). In 2019, a −3 dB modulation bandwidth of ~1.2 GHz was demonstrated from microrod-based LEDs on a sapphire substrate, higher than in any c-plane GaN based LED. A comparison of the −3 dB bandwidth of a planar m- and c-plane LED with a microrod LED is shown in Figure 8c as a function of injection current [1]. Due to the reduced electron–hole wavefunction overlap, the c-plane LED has the smallest bandwidth at low current densities. The bandwidth increases with current due to the screening of the internal field. The m-plane based LED has the highest bandwidth due to the lack of the QCSE and, hence, a high overlap of the electron and the hole wavefunction. The bandwidth of the microrod LED is controlled by both the c-plane (at low injection conditions) and the m-plane (at high injection conditions) of the microrods. When operating the microrod LED at higher injection conditions, the nonpolar planes dominate emission and bandwidth, finally reaching a −3 dB bandwidth above 1 GHz.

## 6. Summary

In summary, we reviewed the progress of bottom-up core–shell InGaN/GaN LEDs from the perspective of device processing and analysis. While the properties of the microrod LEDs are very promising with the potential of overcoming some limiting challenges of planar LEDs, device processing and characterization must be further improved. EL characteristics show changes in the emission color with increasing injection conditions, which is attributed to the inhomogeneous In incorporation in the microrod. Nevertheless, operating microrod LEDs with good efficiencies have been realized, and even white microrod LEDs have been reported. Flexible microrod LEDs have been demonstrated, and microrod LEDs have been shown to exhibit a great potential for high-frequency applications.

## Figures and Tables

**Table 1:** Comparison of electro-optical characteristics (EQE, rectifying value I^+^_(5 V)_/I^−^_(5 V)_) for a given current density (for a given current) from InGaN/GaN core–shell microrod LEDs.

EQE (%)	@ Current Density (A/cm^2^)	Rectifying Value * I^+^_(5 V)_/I^−^_(5 V)_	Additional Information	Year [Reference]
3	40	5	-	2016 [3]
9.3	14.6	17	flexible phosphor-converted white LED	2016 [41]
12.6	14.4	18	blue pump LED	
10	40	153	estimated EQE	2016 [21]
4.8	100 mA	-	-	2017 [42]
8.3	260	85	estimated EQE under room-temperature pulsed operation	2018 [14]
0.63	300 mA	-	flexible phosphor-converted white LED	2019 [4]
2.63	300 mA	-	violet-blue pump LED	

* Rectifying value calculated from published I–V curves.

## Data Availability

No new data were created or analyzed in this study.
